# Cardiac lymphoma requiring surgical resection

**DOI:** 10.1002/jha2.473

**Published:** 2022-05-23

**Authors:** Tomoyo Kubo, Yoshimitsu Shimomura, Yuya Nagai, Takayuki Ishikawa

**Affiliations:** ^1^ Department of Haematology Kobe City Medical Centre General Hospital Kobe Japan

1

A 62‐year‐old woman was admitted to the emergency department with fever, cough, dyspnea, and weight loss of 5 kg in 6 months. Computed tomography revealed numerous lung nodules, masses in both breasts, multiple enlarged lymph nodes, and large low‐density areas in the right atrium. Transthoracic echocardiography showed a 35‐mm hypoechoic mass in the right atrium, extending like a stalk from near the superior vena cava (SVC), which moved significantly with the heartbeat (Figure 1). Breast biopsy revealed a diffuse large B‐cell lymphoma (DLBCL). Intracardiac tumor resection was performed before chemotherapy to prevent tumor embolism or valvular obstruction. The surgery was performed through a midline sternal incision. When the right atrium was incised with extension in the SVC direction, the inside of the right atrium was filled with a tumor with good mobility (Figure 2). The tumor was firmly adherent near the junction of the right atrium and the SVC. Subsequent chemotherapy was expected to shrink the tumor; therefore, the tumor was carefully resected so that the sinus node could remain. The resected tumor consisted of a 35‐mm‐large mass and the portion adherent to the right atrium, which was removed in pieces. Histologic analysis of the resected tumor revealed DLBCL. The patient was started on chemotherapy for stage IV DLBCL 10 days after surgery. She has achieved complete remission, and no arrhythmia or loss of cardiac function has occurred. DLBCL treatment is mainly systemic chemotherapy and, in some cases, irradiation. However, antecedent urgent surgery may be required in some cases when the tumor could cause life‐threatening events [1, 2]. Our patient had no obvious sign of heart failure, but her tumor occupied most of the right atrium and protruded into the right ventricle during diastole with great mobility. There were concerns that the chemotherapy would detach the mass in the right atrium from the SVC and cause tumor embolization or entrapment of the tricuspid valve. Therefore, we decided to perform intracardiac tumor resection prior to chemotherapy. In addition, the goal of surgery was not complete tumor resection but prevention of fatal cardiovascular events; therefore, we tried to preserve normal cardiovascular tissue. We were able to successfully preserve the sinus node at the site of tumor adhesion and the patient has been free of arrhythmia and cardiac dysfunction since the surgery.

**FIGURE 1:**
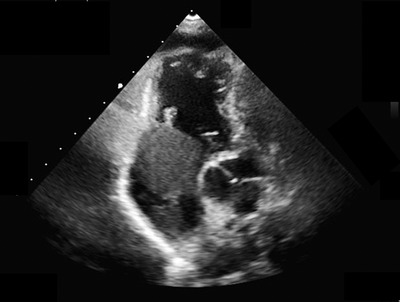
Transthoracic echocardiography revealed tumor filling in the right atrium

**FIGURE 2:**
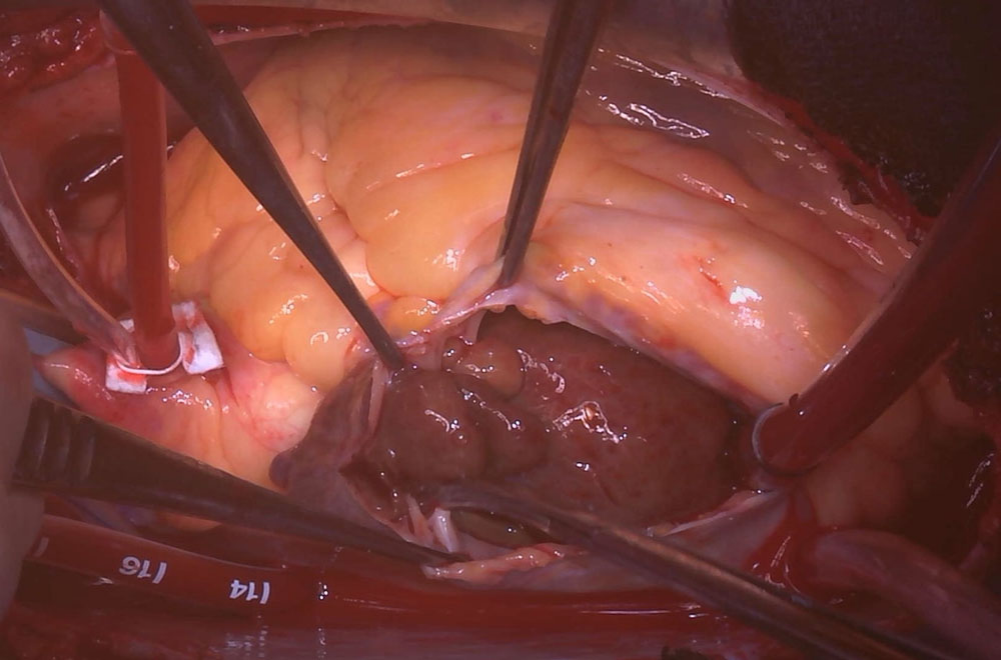
Intraoperative photograph. The right atrium was incised and showed a 35 mm tumor inside

## FUNDING

The authors received no specific funding for this work.

## CONFLICTS OF INTERESTS

The authors declare they have no conflicts of interest.

## ETHICS APPROVAL AND CONSENT TO PARTICIPATE

Publication of this case report does not require approval from the institutional review board of our hospital.

## INFORMED CONSENT STATEMENT

Written informed consent for publication was obtained from the patient.
